# Arriving at SKINTED (Surgery of the Knee, Injury to the Infrapatellar Branch of the Saphenous Nerve, Traumatic Eczematous Dermatitis): A Case Report

**DOI:** 10.7759/cureus.54307

**Published:** 2024-02-16

**Authors:** KavyaDeepu R M, Mohnish Sekar

**Affiliations:** 1 Dermatology, Chettinad Hospital and Research Institute, Chennai, IND; 2 Dermatology, Venereology and Leprosy, Karpaga Vinayaga Institiute of Medical Sciences and Research Centre, Chengalpattu, IND

**Keywords:** immunocompromised district, tkr, autonomic denervation dermatitis, saphenous nerve, skinted

## Abstract

Surgery of the knee, injury to the infrapatellar branch of the saphenous nerve, traumatic eczematous dermatitis (SKINTED) is a postsurgical localized dermatitis specifically linked to total knee arthroplasty (TKA). It is due to autonomic denervation following surgically inflicted nerve injury. It develops several months to years following a surgical trauma. It is being referred to by various names in the literature. Locoregional immune dysfunction due to lymphatic injury after surgery is the currently accepted theory. It must be distinguished from atopic dermatitis, allergic contact dermatitis/sensitization induced by topical medications or implanted metal hypersensitivity dermatitis, and post-traumatic eczema/dermatitis. We present a case of an elderly female patient in her 50s with dry eczematous lesions over the lateral aspect of the surgical incision over both knees developed three months following bilateral total knee replacement (TKR) done in view of osteoarthritis. The patient responded well to topical corticosteroid and emollient treatment. We have also reviewed the literature to provide an overview of potential concepts of etiopathogenesis described in the literature and to clear up any ambiguity surrounding various labels given to this entity.

## Introduction

The nomenclature SKINTED (surgery of the knee, injury to the infrapatellar branch of the saphenous nerve, and traumatic eczematous dermatitis) itself gives us a description of the type of trauma secondary to what and where. It is eczematous dermatitis occurring secondary to injury to the infrapatellar branch of the saphenous nerve (IPBSN) during total knee arthroplasty (TKA) [1]. Trauma to this nerve causes sensory aberrations in the skin innervated by it that are lateral to the incision; skin eruptions in the hypoesthetic area are rare and usually appear three to six months after surgery [2]. In this article, we describe a case of SKINTED, post-total knee replacement (TKR). We reviewed the literature to summarize plausible theories of etiopathogenesis and to clear any doubt concerning the several titles applied to this entity.

The longest branch of the femoral nerve, the saphenous nerve (SN), supplies sensation to the medial aspect of the knee, leg, and foot. It originates from the femoral nerve within the femoral triangle in the thigh and descends the adductor canal, where it emerges as an infrapatellar branch that goes subcutaneously from the medial to the lateral side below the patella [3]. It is prone to trauma after surgical procedures around the knee, primarily TKR [4].

Besides being subcutaneous, the course of its anatomy has been shown to exhibit variations, rendering the IPBSN extremely vulnerable to iatrogenic insult. It has been estimated that up to 70% of cases involving midline incisions for these surgeries result in injury to the IPBSN [5].

Anatomical differences in this nerve exist between patients and between both the lower extremities in the same patient, which could account for the possibility that not all TKR patients experience this eruption [6].

## Case presentation

**Figure 1:**
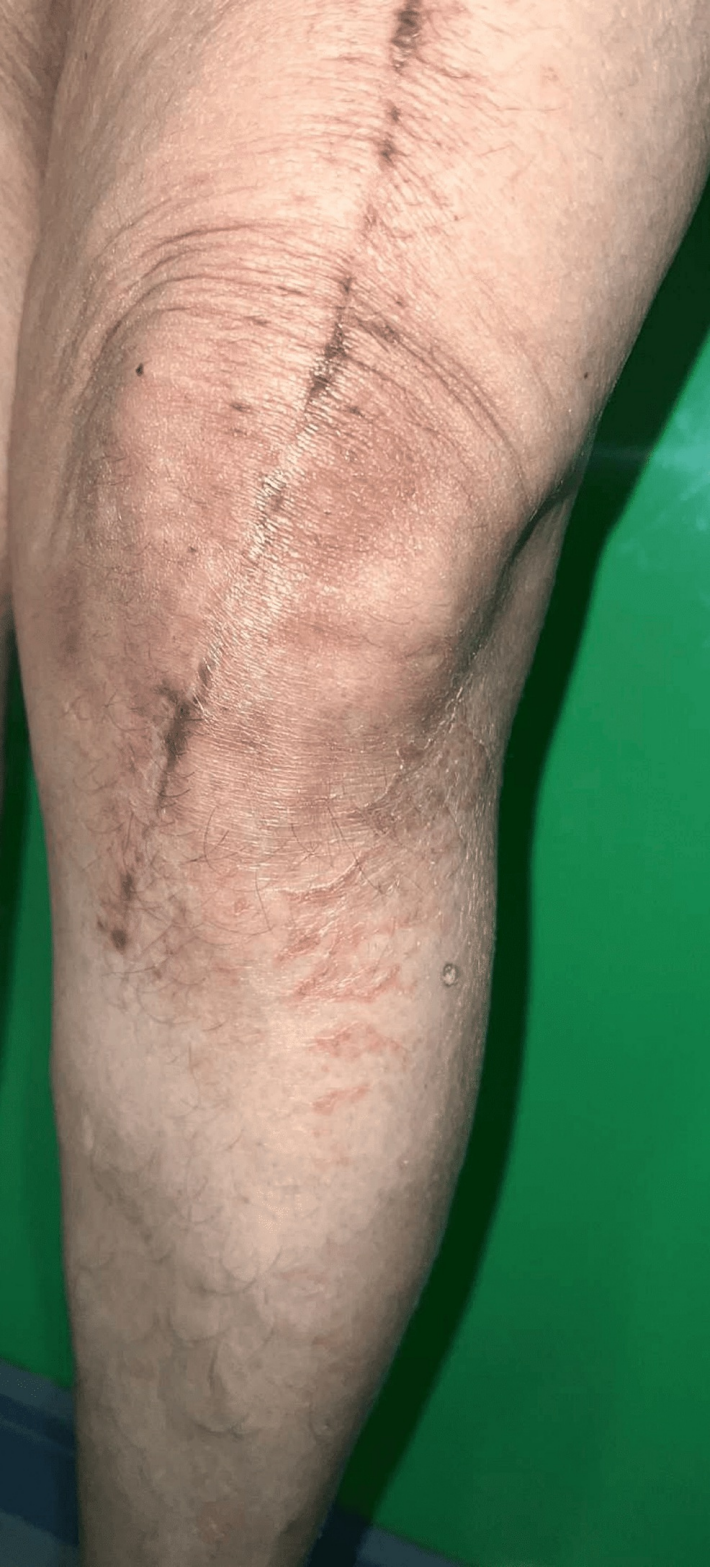
Erythematous plaques with scaling and xerosis noted lateral to incision over left knee.

**Figure 2:**
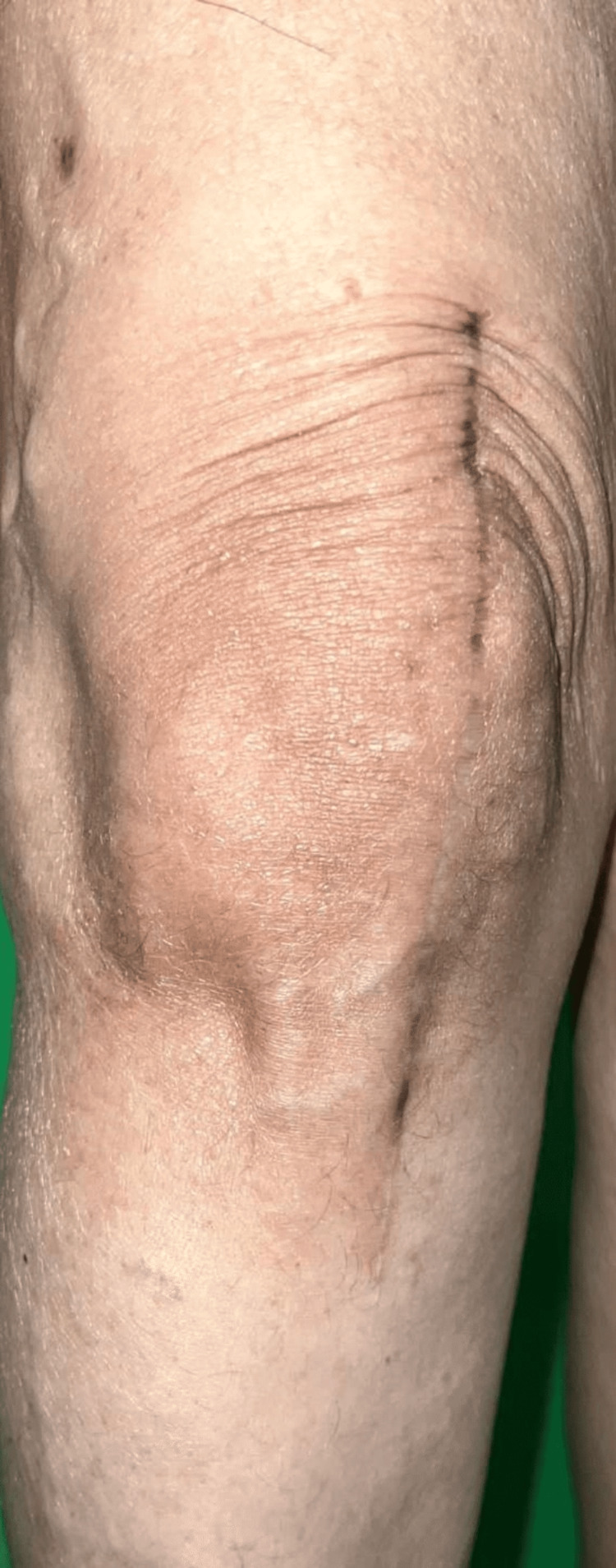
Minimal area of erythematous plaque with scaling noted over right knee.

We report a case of an elderly female patient aged 53 years old who presented to us with non-pruritic reddish, scaly skin lesions over both knees, which appeared three months after a TKR indicated for bilateral knee osteoarthritis. The patient gave a history of numbness of skin over and around the involved area of dermatitis. There was no history of swelling, stiffness, pain or difficulty in movements at the knee joints. The patient was not a known case of preexisting dermatological diseases, including atopic dermatitis and other systemic comorbidities like hypertension or diabetes. There is no history of similar lesions elsewhere over the body with no history of topical medicaments, disinfectant usage, or dressing over the affected area, and no history of metal hypersensitivity or reported dermatitis in the past. Clinically, the lesions were symmetrically distributed on both knees, not crossing the midline surgical incisional line, and were irregular erythematous plaques with scaling and xerosis that suggested asteatotic eczema (Figures 1, 2).

The lesions across the left knee were more prominent than the right knee. There was no swelling, local rise of temperature or tenderness elicited over both the joints. Sensory examination showed hypoesthesia to light touch over the affected site over both limbs. Allergic contact dermatitis was ruled out based on the history of not using any contact allergen like disinfectant, topical ointment, or dressing application. As the eczematous lesions involved areas adjoining the surgical incision site and the rest of the cutaneous surface were normal, we ruled out atopic dermatitis or age-related changes, and the patient was not a known case of other comorbidities like diabetes and hypertension. Metal hypersensitivity was ruled out as there was no history of swelling, stiffness, difficulty in movements or pain at the rest of both knees and the absence of swelling or tenderness elicited over the affected areas on clinical examination, and the rash was non-pruritic.

**Figure 3:**
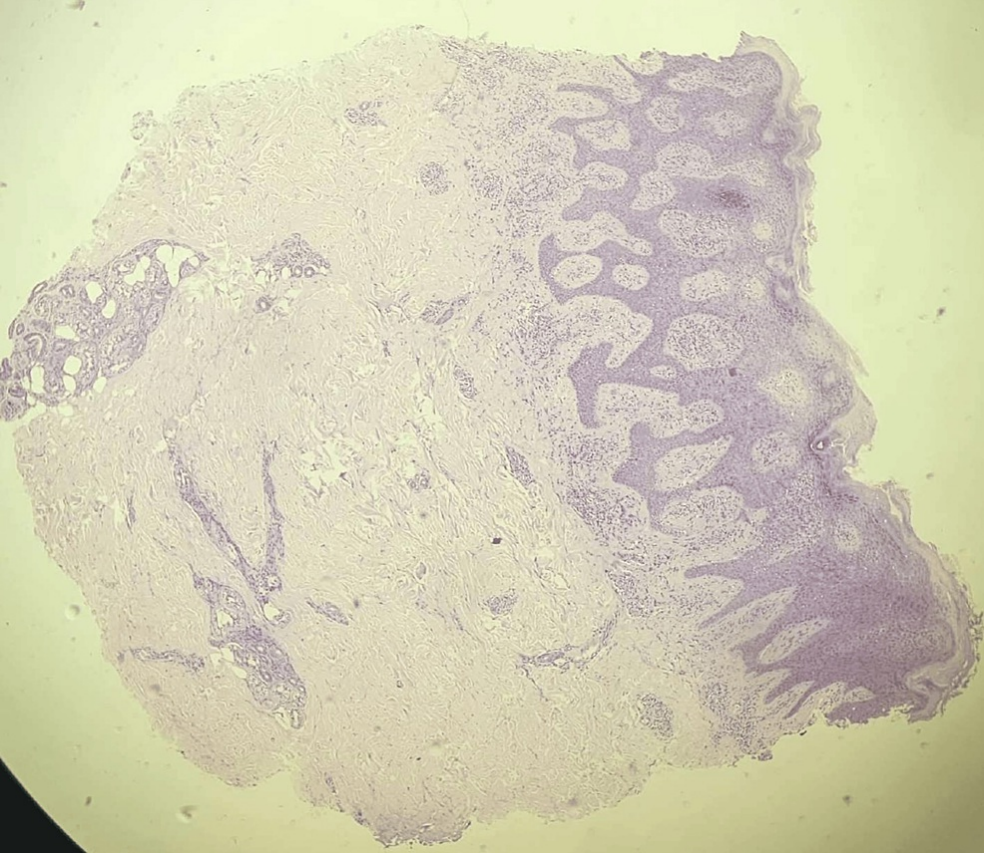
Histopathology reveals hyperkeratosis, spongiosis, elongation of rete ridges and dermis showing infiltration by lymphocytes

Skin scraping for gram staining and culture were negative for bacteria and fungi. Histopathological examination revealed hyperkeratosis, spongiosis and exocytosis of inflammatory cells and dermis showing infiltration by lymphocytes and histiocytes with focal edema suggestive of spongiotic dermatitis (Figure 3).

Potent topical steroid (betamethasone dipropionate cream 0.05%) and topical emollients were prescribed for two weeks, after which the patient showed improvement and was advised to continue emollient for maintenance. No lesions were noted during the three-month follow-up to check on recurrence, but the sensory deficit over the affected area has not improved.

## Discussion

Carr et al. (1981) and Bart et al. (1983) were the first to report dermatitis at the operative site after vein graft harvesting during cardiac surgery [6,7]. Eczematous dermatoses at surgical sites include post-traumatic eczema, neuropathic dermatitis or autonomic denervation dermatitis (ADD), and SKINTED [8]. Eczematous lesions have been hypothesized to be related to the degree of damage to the nerve. For example, a larger incision, along with nerve-crushing injury to some degree from retraction during TKR, may result in a higher risk of nerve injury and cutaneous lesions than a comparatively smaller incision in other knee surgeries [9]. Regarding the controversy surrounding these terms, commonly used interchangeably, a recent literature review shows that a new nomenclature for all TKR cases is SKINTED [1]. A few authors have attempted to differentiate these entities from each other as described in Table 1 [10,11].

**Table 1:** Differentiation between these entities. ADD- Autonomic Denervation Dermatitis, SKINTED- Surgery to Knee, Injury to the infrapatellar branch of the saphenous nerve, traumatic eczematous dermatitis [10,11].

Clinical features	ADD	SKINTED	Post traumatic eczema
Terminologies suggested by	Madke et al. [11]	Verma et al. [1]	Mathios et al. [2]
Appearance of lesions	Months to years following surgery	Months to years after surgery involving knee	Three to four weeks after the trauma
Nature of the trauma	Surgery	Surgery involving knee	Trauma (thermal/chemical/mechanical)
Location	At surgical site	At the surgical site over knee	At traumatic site
Stage of wound healing	Healed surgical site	Healed surgical site	Wound in healing phase
History of atopy	No	No	May be present
Contact allergy	No	No	No
Mechanism	Autonomic disturbance following dermal nerve transections	Autonomic disturbance following dermal nerve transections	Trauma inciting inflammatory response (Koebner)
Associated features	Xerosis & anesthesia/Hypoesthesia In the affected area	Xerosis & anesthesia/Hypoesthesia In the affected area	Variable xerosis & hypoesthesia, depending upon the nature of trauma
Recurrence	Persistent/recurrent	Persistent/recurrent	Recurrent for years

There is a specific entity known as sympathetic dysfunction dermatitis, which is an under-reported side effect following revascularization or replantation surgery that arises from the extremity’s denervation [12]. The exact pathogenesis of skin eruptions occurring in patients over the operated sites remains speculative, and several studies have attempted to explain the underlying pathophysiology which is described below:

Evolution of concepts in literature

Dysfunction of the Autonomic Nervous System

Skin inflammation results from an interplay between autonomic and sensory neurons, which modulates the inflammatory response. Cutaneous inflammation may occur in patients who have had amputations because of a loss of autonomic and sensory nerve modulatory function [13]. Several studies have highlighted the link between the development of skin lesions and autonomic dysfunction. These studies have revealed that patients with atopic dermatitis, psoriasis, lichen planus, and Behcet’s disease illness have sympathetic dysfunction [14-17].

Satku et al. were the first to report dermatitis following TKR in 1993 and suggested autonomic dysfunction and sensory disturbance as contributing, but not exclusive, etiological factors in the development of dermatitis [18]. Autonomic nerve fibres significantly influence cutaneous vasomotor activity, blood flow, and sweat gland function. The alteration results in aberrant keratinocyte proliferation, differentiation, and functioning, triggering these eruptions [11]. Proper functioning of sebaceous glands, sweat glands, and blood flow in the skin maintains the barrier function. One factor contributing to the development of dermatitis is impaired sudomotor activity [8].

Sweat gland stimulation is by sudomotor activity, mediated by unmyelinated C fibres. When these fibres are affected, the skin becomes dry and itchy, which increases the risk of dermatitis over the affected site [14]. A similar outcome is seen with sebaceous glands, which will not secrete sebum-derived lipids, and the skin will lose water and become xerotic, which increases the risk of developing eczema [19]. These autonomic nerve fibres become denervated as a result of traumatic dermal nerve transections brought on by skin incisions during surgery. “Trophoneurosis” is the word used to describe altered cutaneous architecture and physiology after injury to peripheral nerves [8]. Another possible explanation for asteatotic eczema in hyperesthetic skin is a defect in forming inter-corneocyte lipids. Defects in releasing ceramide from the lamellar bodies result in improper water barrier formation. The scar tissue formed post-surgery may impact the role of the granular layer, which is protective and lubrication of keratin and the cutaneous ability to transport substances [19]. This explanation is in line with that provided by Webster et al. who also proposed that postoperative venous stasis following trauma might constitute a risk factor for localized venous congestion and may also result in cutaneous oedema, pigmented purpura resembling inflammatory dermatitis and non-immune bullous eruption [20].

Concept of Wolf’s Isotopic Response

Tüzün et al. defined it as the occurrence of a new skin disorder at exactly the same site as another one, already healed and unrelated [21]. A new dermatosis at the same site is the result of imbalance of pro- and anti-inflammatory peptides such as substance P, bradykinin, serotonin, vasoactive intestinal peptide (VIP), calcitonin gene-related peptide (CGRP), and alpha-melanocyte-stimulating hormone (MSH), which are released in response to an initial herpes zoster virus infection or non-viral injury (surgical trauma to IPBSN in our case). These mediators also induce aberrant T-cell activation, which results in the production of more cytokines and the establishment of a positive feedback loop. Due to local vascular alterations, this limits the immune response to a localized and specific site and increases the recruitment of T cells, which causes the emergence of a new cutaneous disease at the exact location [22]. The concept of locus minoris resistentiae is comparable to this, described as an area of the body that offers lesser resistance to the disease than the rest of the body [23].

New Terminology of SKINTED

Verma et al. coined the abbreviation SKINTED to characterize a localized dermatitis that develops in an area of hypoesthesia lateral to the TKR incision. Damage to the superficial nerves probably incites an eczematous process by weakening the ability of the epidermis to act as a barrier by increasing trans-epidermal water loss (TEWL) and causing xerosis, which then manifests as pruritic eczematous dermatitis. In addition to playing as a defence barrier, the epidermis is involved in the secretion of substance P and acetylcholine. It also controls inflammation, and their absence from an injury might trigger inflammation [1].

Sharquie et al. hypothesized the role of numerous neuropeptides released from the nerve terminals at the time of nerve regeneration. These neuropeptides, which include substance P, VIP, CGRP, and neurotensin, control the presentation of epidermal antigens. These neuropeptides, such as substance P, are also involved in and regulate both immediate and delayed-type cutaneous hypersensitivity reactions [24].

Concept of Immunocompromised District 

All forms of cutaneous scars are known to be susceptible to the development of dysimmune reactions, infections and malignancies. The term “immunocompromised cutaneous district (ICD)” has recently come to encompass the many underlying mechanisms [25]. 

Immunocompetent individuals might develop this sectorial immune deficiency, which is limited to variously injured skin regions, recently referred to as ICDs. This region of ICD is more susceptible than other parts of the body due to inherited or acquired factors. Its vulnerability primarily stems from a local dysregulation of immunological control, which frequently promotes (but occasionally impedes) the local emergence of cutaneous eruptions or disorders associated with immunity. This theory is comparable to the former “locus minoris resistentiae” concept described above [21-23]. The cause is multifactorial and may include previous trauma (including radiation, surgery, burns, and amputations), chronic microscopic or macroscopic lymphatic stasis, healed herpes infections, immunizations, paralytic stroke, or poliomyelitis putting forth minimal resistance. Pathomechanisms include injury to sensory nerve fibres that produce immunopeptides, locally altered lymphatic drainage that inhibits the trafficking of immunocompetent cells, or both. Consequently, any surgical incision, from the time of the procedure to years later, can act as an ICD and lead to the development of not only eczema but also other cutaneous diseases [26].

## Conclusions

While injury to IPBSN is rather prevalent following knee surgery, cutaneous lesions only occur in a few patients. Out of all the studies included in the literature, only eight patients had involvement of both knees, which is uncommon. Diagnosis of SKINTED is clinical, mainly based on the appearance of lesions effectively treated with topical corticosteroids. The long-term course of this condition remains uncertain. Patients with this condition may experience frequent relapses and remissions. Thus, it is necessary to counsel them on the value of using emollients regularly to preserve the integrity of the skin barrier. Several hypotheses exist explaining the pathophysiology of SKINTED; however, none of them has been shown to be precise or conclusive. Hence we recommend more research to comprehend the pathophysiology behind the cutaneous eruptions following TKR completely.
